# Ubiquitin-specific protease as the underlying gene biomarker for aortic stenosis

**DOI:** 10.1186/s12944-020-01299-3

**Published:** 2020-05-29

**Authors:** Yin Yang, Lian-qun Wang, Bo-chen Yao, Zhi-gang Guo

**Affiliations:** Department of Cardiac Surgery, Tianjin Chest Hospital, No. 261 South Taierzhuang Road, Jinnan District, Tianjin, 300222 China

**Keywords:** Aortic stenosis, Ubiquitin-specific protease 14, Gene biomarker, Bioinformatics analysis, Lipid metabolism

## Abstract

**Background:**

Aortic stenosis is a common heart valvular disease whose pathological processes include an inflammatory reaction and lipid accumulation. However, its detailed pathogenesis is yet to be completely elucidated. Therefore, it is of great significance to further explore the molecular mechanisms of aortic stenosis.

**Methods:**

Four datasets were downloaded from the Gene Expression Omnibus (GEO) database. Firstly, the differently expressed genes (DEGs) were screened between control and aortic stenosis samples. Secondly, weighted gene co-expression network analysis (WGCNA) was performed to find the highly relevant gene modules. Enrichment analysis and protein-protein interaction (PPI) networking were also performed, then Cytoscape was used to identify hub genes. Finally, the six participants (3 control participants and 3 patients with aortic stenosis) were recruited at the Tianjin Chest Hospital. In order to verify the expression level of USP14, several molecular experiments were performed, including hematoxylin-eosin (HE) staining, immunohistochemistry, immunofluorescence technology, real time-quantitative polymerase chain reaction (RT-qPCR), and western blotting.

**Results:**

A total of 9636 DEGs were found between the control and aortic stenosis samples. The DEGs were mainly enriched in the autophagy-animal, cellular lipid catabolic process, apoptosis, and glycoside metabolic process categories. Eleven hub genes were identified via four different algorithms. Following verification of the patient samples, Ubiquitin-specific protease 14 (USP14) was found to be displayed at higher levels in the aortic stenosis samples.

**Conclusion:**

USP14 might be involved in the occurrence and development of aortic stenosis, so it would be a molecular target for early diagnosis and specific treatment of aortic stenosis. There is a significant association between the high expression of USP14 and aortic stenosis, indicating that this gene may be a genetic risk factor for aortic stenosis.

## Background

Aortic stenosis (AS) is a common heart valvular disease [1]. In recent years, the incidence of AS has been on the rise. Apart from its degenerative, congenital (bicuspid valve), and inflammatory causes, AS might also be caused by rheumatic heart disease and other less common diseases, such as Paget’s disease, Fabry disease, and lupus erythematosus. The diagnosis of aortic stenosis is based on an echocardiographic assessment [2]. The valve area is 3–4 cm^2^ in healthy adults. In general, AS patients do not exhibit clinical symptoms until the valve area is less than 1.0 cm^2^. At that point, the extra work required to open the valve prevents the heart from functioning properly. Currently, age-related degenerative aortic stenosis has become the most common cause of aortic stenosis in adults. The prevalence of aortic stenosis increases with age, averaging 0.2% in people aged 50 to 59 and 9.8% in people aged 80 to 89 [3].

The pathological process of aortic stenosis includes an inflammatory reaction, lipid accumulation, angiotensin converting enzyme activation, and macrophage and T lymphocyte infiltration. The activity of the valve is limited due to the deposition of calcium at the base of the valve, which leads to aortic stenosis. Aortic valve calcification is similar to coronary heart disease and has a high correlation with coronary artery calcification. Hypertension, dyslipidemia, diabetes, and smoking are the risk factors [4, 5].

Aortic stenosis is a slowly progressing disease. Patients with aortic stenosis have a long asymptomatic period; those without symptoms need no treatment but should follow-up regularly. However, when symptoms appear or obstruction becomes very severe, rapid diagnosis and treatment is essential [6]. At present, the treatments for aortic stenosis include surgical aortic valve replacement (AVR) and transcatheter aortic valve implantation (TAVI) [7]. In fact, sometimes patients are referred to AVR after the myocardium has started to decompensate, which might cause new tissue damage and lead to serious complications. Thus, early diagnosis and prevention are needed. In addition, TAVI is also associated with paravalvular aortic regurgitation, stroke, vascular complications, and the need for a new pacemaker.

Therefore, it is of great clinical significance to further explore the molecular mechanisms of aortic stenosis, in order to find molecular targets for early diagnosis, prevention, and specific treatment. Microarray technology can use genomic data to explore and mine disease-related differentially expressed genes. At present, it is an effective method to explore the pathogenesis of diseases [8]. To meet these goals, this paper uses microarray technology to mine the differentially expressed genes between stenotic valves and normal aortic valves, and to identify the hub genes of aortic stenosis. Results are further verified by molecular experiments.

## Methods

### Data from the GEO database

On October 16, 2019, the key words “(aortic valve) AND calcified” were set to detect the datasets, using a filter of “expression profiling by array.” There were five inclusion criteria: a sample number of more than ten per dataset (samples of less than ten were excluded), data from *Homo sapiens* (data from other species were excluded), a series entry type, expression profiling by array (data using methylation profiling by array were excluded), and a diagnosis of aortic stenosis caused by the aortic valve sclerosis or calcification (data from mitral valve stenosis diagnoses were excluded).

The study obtained the transcriptome expression profiles GSE12644 (GPL570 [HG-U133_Plus_2] Affymetrix Human Genome U133 Plus 2.0 Array), GSE51472 (GPL570 [HG-U133_Plus_2] Affymetrix Human Genome U133 Plus 2.0 Array), GSE83453 (GPL10558 Illumina Human HT-12 V4.0 expression beadchip) and GSE88803 (GPL6244 [HuGene-1_0-st] Affymetrix Human Gene 1.0 ST Array [transcript (gene) version]) from the GEO database (Table 1).

**Table 1:** A summary of aortic valve stenosis microarray datasets from different GEO datasets

	Series	Platform	Affymetrix GeneChip	Samples	Normal	Calcification
1	GSE12644	GPL570	[HG-U133_Plus_2] Affymetrix Human Genome U133 Plus 2.0 Array	20	10	10
2	GSE51472	GPL570	[HG-U133_Plus_2] Affymetrix Human Genome U133 Plus 2.0 Array	15	5	10
3	GSE83453	GPL10558	Illumina HumanHT-12 V4.0 expression beadchip	27	8	10
4	GSE88803	GPL6244	[HuGene-1_0-st] Affymetrix Human Gene 1.0 ST Array [transcript (gene) version]	10	5	5

### Screening of DEGs

The four GEO series were merged and normalized by the R packages sva and limma. The DEGs were screened by R package limma; the cut-off criteria were *P* value < 0.05 and log [Fold Change (FC)] ≥ 100 or ≤ − 100.

### WGCNA analysis

WGCNA is an algorithm for mining module information from chip data, which can describe the patterns of genes between microarray samples and find highly relevant gene modules. In this study, WGCNA analysis was conducted by the R package WGCNA. The lowest thresholding power for the scale-free topology fit index was 0.9. The ME-Diss Thresh was set at 0.1 to merge similar modules.

### Functional annotation of DEGs

Gene Ontology (GO) (see http://geneontology.org/docs/introduction-to-go-resource/, version 10.5281/zenodo.2529950, released January 1, 2019) analysis is an ontology widely used in bioinformatics analysis, containing three aspects of biology: biological processes (BP), cellular components (CC), and molecular functions (MF). The Kyoto Encyclopedia of Genes and Genomes (KEGG) (https://www.genome.jp/kegg/docs/relnote.html, version 92.0, released October 1, 2019) analysis can provide specific pathways and link genomic information with higher-order functional information. Gene Set Enrichment Analysis (GSEA) (version 4.0.2) is a computational method that can execute GO and KEGG analysis with a given gene list. Metascape (http://metascape.org/gp/index.html#/main/step1, released August 14, 2019) is an online analysis tool providing a comprehensive gene list annotation and analysis resource. In this study, the GO and KEGG analysis of MEdarkgrey model DEGs were performed by GSEA and Metascape. The *p*-value cutoff was 0.05, with Min Overlap = 3 and Min Enrichment = 1.5.

### Construction and analysis of the PPI network

STRING (http://string-db.org, Version 11.0), an online database, can predict and provide a PPI network after importing the MEdarkgrey model DEGs. For this analysis, all data sources were considered. The minimum required interaction score was medium confidence (0.400). Cytoscape (Version 3.7.2) is an analysis tool used to provide biological network analysis and two-dimensional (2D) visualization for biologists. The algorithms which were used for network analysis included a Betweenness algorithm, Radiality algorithm, EcCentricity algorithm, and Closeness algorithm. In this study, the PPI network and hub genes were constructed and analyzed by the STRING database and Cytoscape.

### Identification of hub DEGs associated with cardiovascular diseases

The comparative toxicogenomics database (CTD) (http://ctdbase.org/, released November 6, 2019 [15938]) is a web-based database that can predict the relationship between a gene/protein and disease. Operation instructions are available at http://ctdbase.org/help/. In this study, the relationships between gene products and heart diseases were analyzed by this database.

### MiRNA of hub DEGs prediction

TargetScan (www.targetscan.org, released March 2, 2018) is an online database that performs predictive analysis and identifies possible mechanisms for co-regulating the expression of hundreds of genes expressed in different cell types. In this study, TargetScan was used to screen for miRNAs that regulate the hub DEGs.

### Functional annotation of miRNA

DIANA-miRPath v3.0 (http://www.microrna.gr/miRPathv3, version 3) is an online analysis tool suite dedicated to conducting functional and pathway enrichment analysis for miRNA. In this study, GO and KEGG pathway enrichment analysis were performed using miRPath (*P* < 0.05).

### Statistical analysis

The analyses in this study were performed using Perl, R (version 3.5.3), and SPSS 20.0 (SPSS Inc., Chicago, IL, USA). The hub DEGs and their effect on aortic stenosis, based on univariate logistic proportional regression analysis and multivariate logistic proportional regression analysis, were analyzed via SPSS 20.0. Finally, the relative operating characteristic (ROC) curve was provided by SPSS 20.0 and MedCalc software (MedCalc Software Ltd., Ostend, Belgium).

### Participants and ethics

At the Tianjin Chest Hospital, the pathological sections were obtained between 2018 and 2019 from six participants. The inclusion criteria included being between 18 and 80 years old, a diagnosis of aortic stenosis, and a non-surgical history. Patients whose poor cardiac function, pulmonary function, or liver and kidney function made them unable to tolerate surgery were excluded.

The diagnosis of aortic stenosis was based on the preoperative surface echocardiography, intraoperative esophageal echocardiography, and intraoperative findings [6]. The pathological report manifested that a total of 3 participants were diagnosed with aortic stenosis while 3 others were not. The demographic details of the participants are shown in Table 2. The research conformed to the Declaration of Helsinki, and it was authorized by the Human Ethics and Research Ethics Committees of the Tianjin Chest Hospital. Informed consent was provided by all the participants.

**Table 2:** The demographic details of participants

Characteristics			Aortic stenosis		P
			No	Yes	
Sex	Male	4	1(16.7%)	3(50.0%)	0.083
	Female	2	2(33.3%)	0(0.0%)	
Age		6	58.00 ± 4.58	65.33 ± 3.06	0.082
Co-morbidities	No	1	1(16.7%)	0(0.0%)	0.273
	Yes	5	2(33.3%)	3(50.0%)	

Independent-samples T test was used to compare continuous data. Pearson’s chi-squared test was used to compare the categorical data. The co-morbidities in this research included hypertension, diabetes, and coronary heart disease. “No” of “Co-morbidities” presented that the participants did not suffer from any of the above three co-morbidities. “Yes” of “Co-morbidities” presented that the participant suffered from all above three co-morbidities

### Histopathological examination

The aortic valve was dissected and cut into parts. Part of the artery was fixed in a 4% poly-formaldehyde solution and processed for paraffin embedding and sectioning. The aortic valve was cut into 6-μm-thick transverse sections and stained with HE after fixation in a 4% poly-formaldehyde solution embedded in paraffin wax. Then, the immunohistochemistry and immunofluorescence assays were performed to detect the expression level of USP14.

The samples of the aortic valve were taken to make paraffin sections. The paraffin sections were deparaffinized to water, sealed with H_2_O_2_, and washed with double distilled water. After antigen retrieval, immunohistochemistry was used to detect the USP14. The specific detection steps were performed according to the instructions of the VECTASTAIN® Elite® ABC Kit (Vector Laboratories Inc., 30 Ingold Road Burlingame, California, USA), as follows.

First, the antigen-fixed paraffin sections were washed with phosphate buffer solution (PBS) 2–3 times (5 min/time), then blocked with 10% goat serum (TransGen Biotech Co., LTD, Beijing, China) at 37 °C for 20 min. Filter paper was used to remove the serum. The mouse anti-human USP14 polyclonal antibody (dilution rate = 1:3000, SAB1406778, Sigma, St. Louis, USA) was added dropwise and incubated overnight at 4 °C, washed three times with PBS (5 min/time), and incubated with goat anti-mouse monoclonal antibody (dilution rate = 1:5000, ab205719, Abcam, Cambridge, UK) at 37 °C for 1 h. Diaminobenzidine (DAB) was used for color development (PBS instead of the primary antibody was used as a negative control). Each paraffin section was photographed in 5 fields and counted [9].

### RT-qPCR assay

Total RNA was extracted by the RNAiso Plus (Trizol) kit (Thermofisher, Massachusetts, USA), then reverse transcribed to cDNA. RT-qPCR was performed using a Light Cycler® 4800 System with specific primers for USP14. Table 3 presents the primer sequences. The glyceraldehyde-3-phosphate dehydrogenase (GAPDH) was used as an endogenous control.

**Table 3:** Primers and their sequences for PCR analysis

Primer	Sequence (5′–3′)
ACTIN-hF	CACCCAGCACAATGAAGATCAAGAT
ACTIN-hR	CCAGTTTTTAAATCCTGAGTCAAGC
USP14-hF	TGTGCCTGAACTCAAAGATGCC
USP14-hR	ACTGTCCTTGTTCACCTTTCTCG

### Western blotting analysis

Aortic valve tissues were frozen at -80 °C. Total protein was isolated in a lysis buffer, resolved by 10% sodium dodecyl sulfate polyacrylamide gel electrophoresis (SDS-PAGE), and transferred onto polyvinylidene fluoride (PVDF) membranes by electroblotting. After blocking it with the Odyssey blocking buffer (LI-COR, Lincoln, USA) for 1 h, the membrane was incubated with primary antibodies at 4 °C overnight, and then it was incubated with a horseradish peroxidase-conjugated secondary antibody at 37 °C for 1 h.

Next, the protein bands were scanned and analyzed with the Odyssey scanner [10]. GAPDH was used as an endogenous control. Mouse anti-human GAPDH polyclonal antibody (dilution rate = 1:5000, WH0002597M1, Sigma, St. Louis, USA) was used to detect GAPDH, and the secondary antibody was also goat anti-mouse monoclonal antibody (dilution rate = 1:5000, ab205719, Abcam, Cambridge, UK). USP14 protein was detected using a mouse anti-human USP14 polyclonal antibody (dilution rate = 1:3000, SAB1406778, Sigma, St. Louis, USA). The secondary antibody was goat anti-mouse monoclonal antibody (dilution rate = 1:5000, ab205719, Abcam, Cambridge, UK). The bands were visualized with an enhanced chemiluminescence kit (Millipore, Billerica, Massachusetts, USA) and analyzed with Image-Pro Plus 6.0 (Media Cybernetics Inc., Chicago, USA).

## Results

### Identification of DEGs

**Fig. 1:**
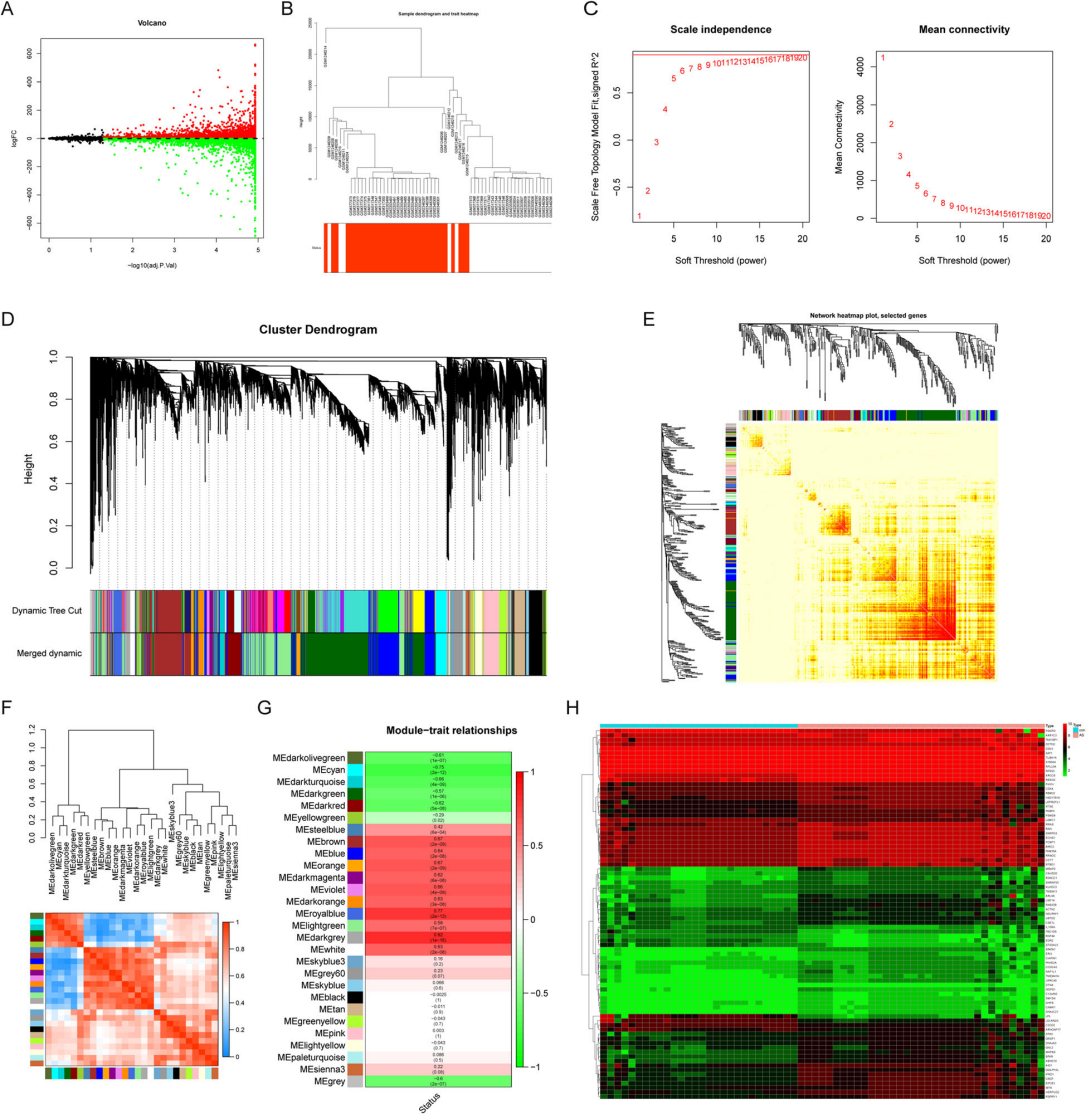
Differential expression analysis and WGCNA analysis of the genes in the merged series. **a** Volcano plots of the genes which are different expression (DEGs) between aortic valve stenosis group and normal group. **b** The cluster of patients with clinical information, red line represents patients with aortic valve stenosis. **c** The lowest power for which scale independence. **d** Repeated hierarchical clustering tree of the 9636 genes.**e** The dendrogram and heatmap of genes. **f** Interactions between these modules. **g** The associations between clinic traits and the modules. **h** Heatmaps of the 83 DEGs in the MEdarkgrey model

The merged series contained 5792 up-regulated genes and 3844 down-regulated genes (Fig.1a).

### WGCNA analysis and attainment of module DEGs

In this study, WGCNA analysis was conducted using the R package WGCNA. The cluster of patients is shown in Fig. 1b. As shown in Fig. 1c, a power value of 8 was the lowest power for which scale independence was below 0.9, so it was selected to produce a hierarchical clustering tree of the 6301 genes (Fig. 1d). In addition, a dendrogram and heatmap were used to quantify module similarity by correlation (Fig. 1e). Interactions between these modules were then analyzed (Fig. 1f). The associations between clinical traits and the modules were identified based on the correlation between module and clinical traits (Fig. 1g). The heat map of the MEdarkgrey model DEGs’ expression is shown in Fig. 1h.

### Functional and pathway enrichment analysis of DEGs

**Fig. 2:**
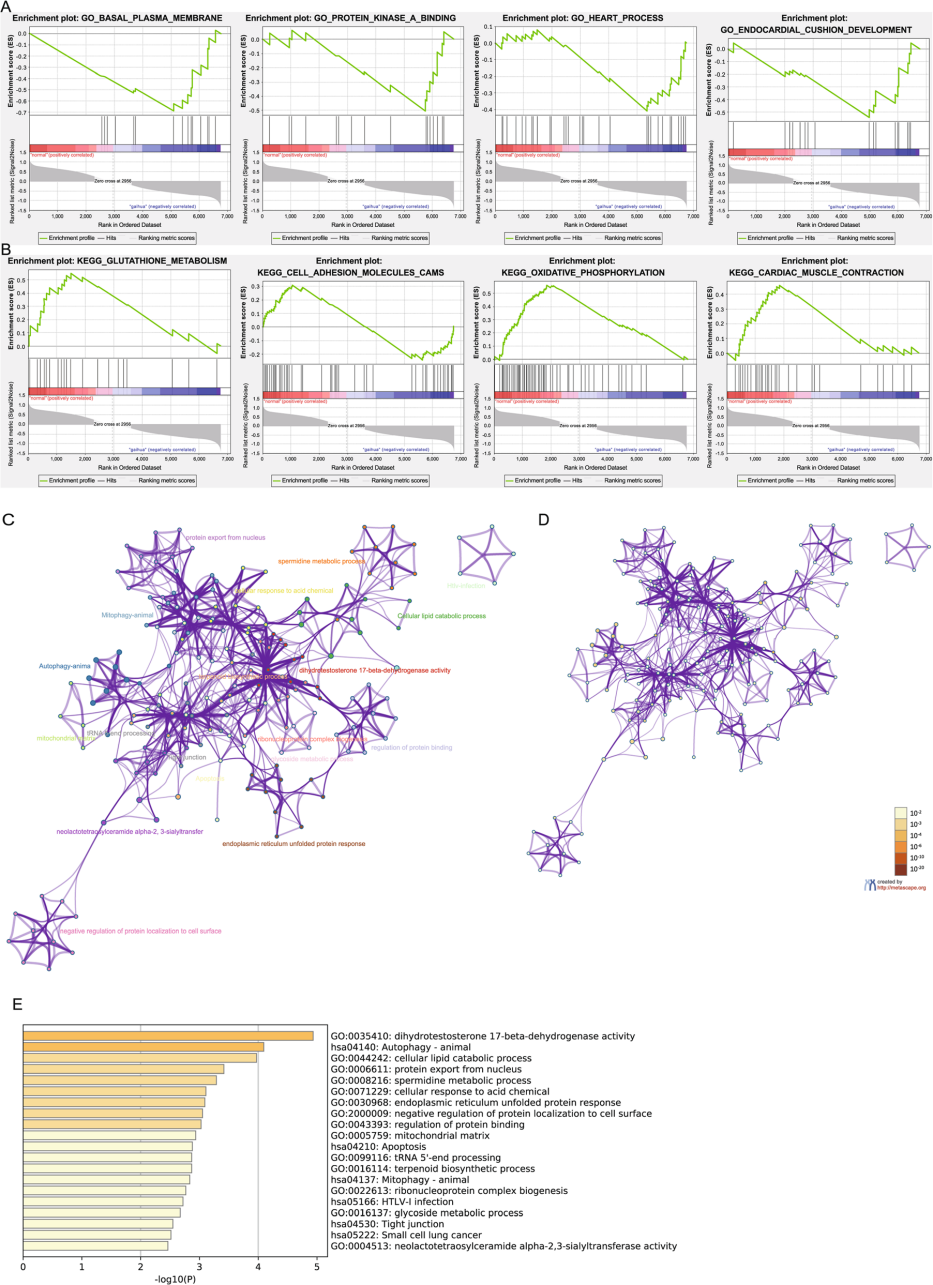
Gene functional enrichment analysis of the MEdarkgrey model DEGs by GSEA and Metascape. **a** Gene ontology (GO) analyses by GSEA. **b** Kyoto Encyclopedia of Genes and Genomes (KEGG) analyses of by GSEA. **c** Enrichment_GO-KEGG_ColorByCluster analyses by Metascape. **d** Enrichment_GO-KEGG_ColorByPValue analyses by Metascape. **e** Enrichment_heatmap_HeatmapSelected GO-KEGG analyses by Metascape

GSEA to DEGs elicited an enrichment of the “heart process” and “cardiac muscle contraction” (Fig. 2a-b, Tables 4 and 5). Metscape analysis of DEGs showed an enrichment in the autophagy-animal, cellular lipid catabolic process, apoptosis, and glycoside metabolic process categories (Fig. 2c-e).

**Table 4:** GO analysis by GSEA

TERM	SIZE	NES	p-val	RANK AT MAX	LEADING EDGE
Up-regulated					
GO_REGULATION_OF_CARDIAC_MUSCLE_CELL_CONTRACTION	18	−1.67584	0.003914	1264	tags = 61%, list = 19%, signal = 75%
GO_HEART_PROCESS	36	−1.50175	0.037109	1395	tags = 42%, list = 21%, signal = 52%
GO_PROTEIN_KINASE_A_BINDING	17	−1.51351	0.019841	977	tags = 47%, list = 14%, signal = 55%
GO_ENDOCARDIAL_CUSHION_DEVELOPMENT	21	−1.55367	0.009862	1768	tags = 52%, list = 26%, signal = 71%
GO_STRUCTURAL_CONSTITUENT_OF_CYTOSKELETON	42	−1.6003	0.00996	1034	tags = 40%, list = 15%, signal = 48%
Down-regulated					
GO_MULTICELLULAR_ORGANISMAL_MACROMOLECULE_METABOLIC_PROCESS	43	1.747944	0.012146	679	tags = 51%, list = 10%, signal = 57%
GO_MULTICELLULAR_ORGANISM_METABOLIC_PROCESS	47	1.695392	0.014344	679	tags = 47%, list = 10%, signal = 52%
GO_CELL_CHEMOTAXIS	76	1.53365	0.031955	1128	tags = 53%, list = 17%, signal = 62%
GO_REGULATION_OF_BONE_REMODELING	18	1.481831	0.01145	916	tags = 50%, list = 14%, signal = 58%
GO_REGULATION_OF_STEROL_TRANSPORT	15	1.481576	0.059289	1346	tags = 53%, list = 20%, signal = 66%

*NES* Normalized Enrichment Score

**Table 5:** KEGG analysis by GSEA

TERM	SIZE	NES	p-val	RANK AT MAX	LEADING EDGE
Up-regulated					
KEGG_ARRHYTHMOGENIC_RIGHT_VENTRICULAR_CARDIOMYOPATHY_ARVC	40	−1.38995	0.071984	1598	tags = 43%, list = 24%, signal = 55%
KEGG_PEROXISOME	37	−1.21825	0.236686	1355	tags = 43%, list = 20%, signal = 54%
KEGG_PEROXISOME	37	−1.21825	0.236686	1355	tags = 43%, list = 20%, signal = 54%
KEGG_GAP_JUNCTION	50	−1.20703	0.208333	1746	tags = 44%, list = 26%, signal = 59%
KEGG_CALCIUM_SIGNALING_PATHWAY	67	−1.12748	0.296813	1585	tags = 36%, list = 23%, signal = 46%
Down-regulated					
KEGG_CARDIAC_MUSCLE_CONTRACTION	32	1.191075	0.318271	1839	tags = 66%, list = 27%, signal = 90%
KEGG_CYTOKINE_CYTOKINE_RECEPTOR_INTERACTION	94	1.251404	0.201942	1695	tags = 55%, list = 25%, signal = 73%
KEGG_GLUTATHIONE_METABOLISM	23	1.371425	0.064327	1499	tags = 61%, list = 22%, signal = 78%
KEGG_OXIDATIVE_PHOSPHORYLATION	76	1.331254	0.211045	1839	tags = 70%, list = 27%, signal = 95%
KEGG_CELL_ADHESION_MOLECULES_CAMS	64	1.131856	0.316602	1047	tags = 39%, list = 16%, signal = 46%

### Construction and analysis of the protein-protein interaction network

**Fig. 3:**
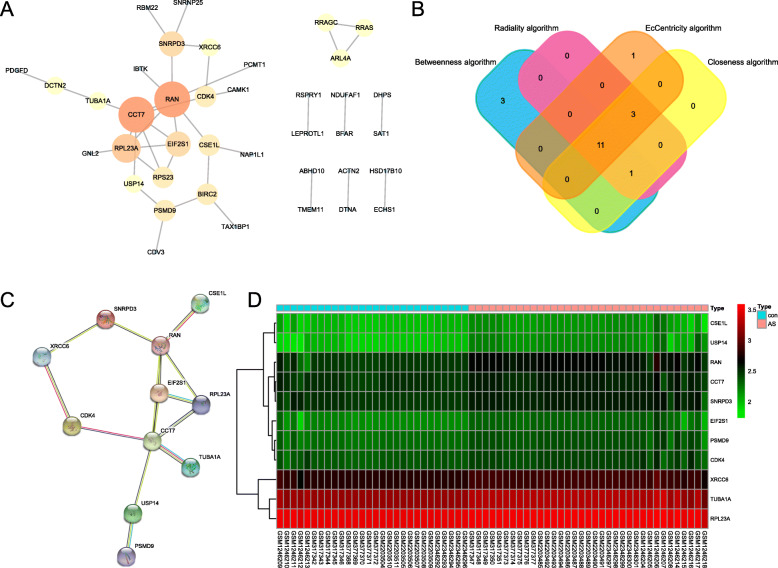
Relationship between DEGs. **a** Protein-protein interaction (PPI) network, the more the number of connections, the larger of the protein. The orange was defined as dark color to map parameters, which represented the high value of protein. The yellow was defined as middle color to map parameters, which presented the middle value of protein. The blue was defined as bright color to map parameters, which represented the low value of protein. The small sizes showed the low values, and the large sizes represented the high values. **b** The common hub genes identified from different algorithm. **c** The common hub genes of protein-protein interaction network. **d** Heat maps of the common hub genes

The PPI network of the DEGs was constructed via the STRING online database and analyzed by Cytoscape software (Fig. 3a). Four different algorithms were employed to identify hub genes and 11 common hub genes were obtained (Fig. 3b). A summary of common hub genes is shown in Table 6. The PPI network of common hub genes is shown in Fig. 3c. The heat map of common hub genes is shown in Fig. 3d.

**Table 6:** A summary of hub genes

Symbol	Description	Funtion
RAN	RAN, member RAS oncogene family	GO:0035281 pre-miRNA export from nucleus
		hsa03008: Ribosome biogenesis in eukaryotes
		hsa03013: RNA transport
CCT7	chaperonin containing TCP1 subunit 7	GO:1904871 positive regulation of protein localization to Cajal body
		GO:1904869 regulation of protein localization to Cajal body
		GO:1904874 positive regulation of telomerase RNA localization to Cajal body
CSE1L	chromosome segregation 1 like	GO:0006606 protein import into nucleus
		GO:0006611 protein export from nucleus
		GO:0051170 import into nucleus
SNRPD3	small nuclear ribonucleoprotein D3 polypeptide	GO:0000387 spliceosomal snRNP assembly
		GO:0008334 histone mRNA metabolic process
		hsa03040: Spliceosome
TUBA1A	tubulin alpha 1a	GO:0097711 ciliary basal body-plasma membrane docking
		hsa04540: Gap junction;
		hsa04210: Apoptosis
USP14	ubiquitin specific peptidase 14	GO:1903070 negative regulation of ER-associated ubiquitin-dependent protein catabolic process
		GO:1903069 regulation of ER-associated ubiquitin-dependent protein catabolic process
		GO:1904293 negative regulation of ERAD pathway
PSMD9	proteasome 26S subunit, non-ATPase 9	GO:0002223 stimulatory C-type lectin receptor signaling pathway
		GO:0002479 antigen processing and presentation of exogenous peptide antigen via MHC class I, TAP-dependent
		GO:0046676 negative regulation of insulin secretion
CDK4	cyclin dependent kinase 4	GO:1904637 cellular response to ionomycin; 12-myristate
		GO:1904628 cellular response to phorbol 13-acetate
		GO:1904636 response to ionomycin
RPL23A	ribosomal protein L23a	GO:0006614 SRP-dependent cotranslational protein targeting to membrane
		GO:0006613 cotranslational protein targeting to membrane
		hsa03010: Ribosome
XRCC6	X-ray repair cross complementing 6	GO:0075713 establishment of integrated proviral latency
		GO:0097680 double-strand break repair via classical nonhomologous end joining
		hsa03450: Non-homologous end-joining
EIF2S1	eukaryotic translation initiation factor 2 subunit alpha	GO:1990737 response to manganese-induced endoplasmic reticulum stress
		GO:0032057 negative regulation of translational initiation in response to stress;process
		hsa04140: Autophagy - animal

### Identification of hub genes

**Fig. 4:**
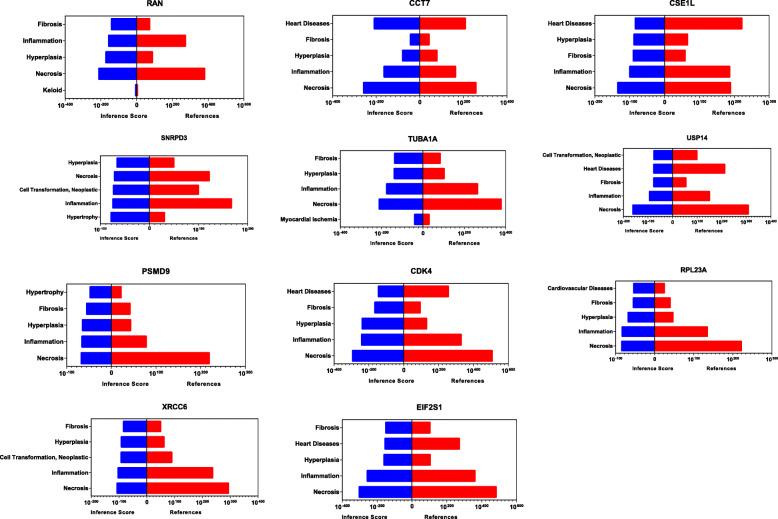
Relationship to aortic valve stenosis group and normal group related to DEGs based on the CTD database. The blue column represented the inference score, and the red column represented the references

The CTD database showed that common hub genes targeted heart diseases. It also highlighted pathophysiological processes such as fibrosis, inflammation, and necrosis, which are relevant in aortic stenosis pathogenesis (Fig. 4).

### Prediction and functional annotation of miRNA associated with hub genes

**Fig. 5:**
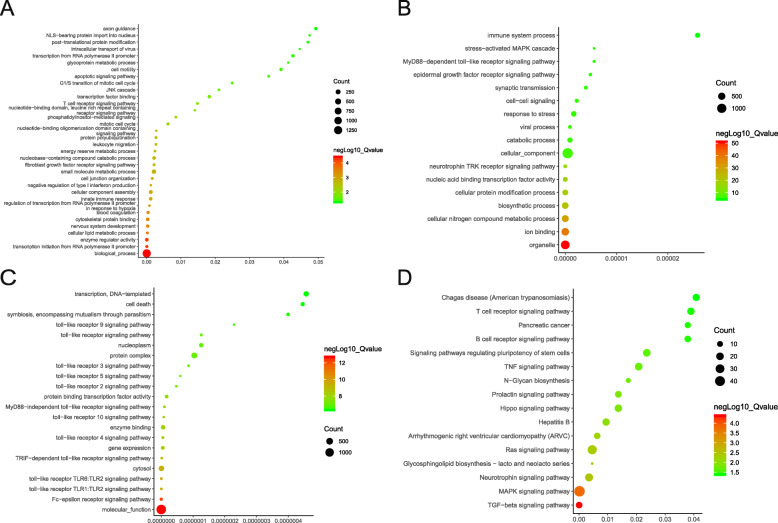
Functional and pathway enrichment analysis of miRNAs which could regulate hub genes. **a** BP analyses (**b**) CC analyses. **c** MF analyses. **d** KEGG analyses of the miRNAs. BP: biological processes, CC: cellular component, MF: molecular functions, KEGG: Kyoto Encyclopedia of Genes and Genomes

The miRNAs that regulate the hub genes were screened out with TargetScan (Table 7). GO and KEGG analyses of the miRNA were performed by DIANA-miRPath. GO and KEGG enrichment analysis to the screened miRNA demonstrated a preponderance of the cellular lipid metabolic process, apoptotic signaling pathway, nucleic acid binding transcription factor activity, toll-like receptor signaling pathway, Hippo signaling pathway, Arrhythmogenic right ventricular cardiomyopathy, and others (Fig. 5).

**Table 7:** A summary of miRNAs that regulate hub genes

	Gene	Predicted MiR		Gene	Predicted MiR
1	RAN	hsa-miR-489-3p hsa-miR-324–5p hsa-miR-205-5p	7	PSMD9	hsa-miR-532-5p hsa-miR-9-5p hsa-miR-30d-5p
2	CCT7	hsa-miR-3607-5p hsa-miR-3194–5p hsa-miR-6858-3p	8	CDK4	hsa-miR-326 hsa-miR-330-5p hsa-miR-483-3p.1
3	CSE1L	hsa-miR-6835-3p hsa-miR-377-3p hsa-miR-19b-3p	9	RPL23A	hsa-miR-892c-5p hsa-miR-506-5p hsa-miR-6074
4	SNRPD3	hsa-miR-330-3p hsa-miR-193a-3p hsa-miR-193b-3p	10	XRCC6	hsa-miR-1207-3p hsa-miR-7843-5p hsa-miR-4632-5p
5	TUBA1A	hsa-miR-15b-5p hsa-miR-497-5p hsa-miR-424–5p	11	EIF2S1	hsa-miR-26a-5p hsa-miR-26b-5p hsa-miR-4465
6	USP14	hsa-miR-543 hsa-miR-4429 hsa-miR-320d

### Logistic regression analysis and identification of USP14

**Fig. 6:**
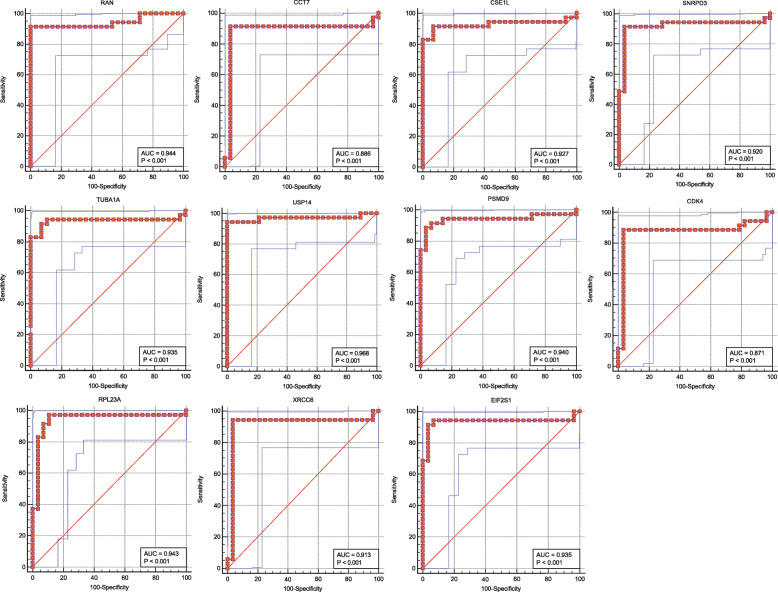
ROC curves of hub genes

The ROC curves of common hub genes are shown in Fig. 6. Likewise, the common hub genes and their effect on aortic stenosis, based on univariate logistic proportional regression analysis, are shown in Table 8 and those based on multivariate logistic proportional regression analysis in Table 9. Of the eleven potential biomarkers, USP14 was chosen, partly due to its higher odds radio (OR) calculated after multivariate logistic regression analysis (Table 9).

**Table 8:** The hub genes and their effect on aortic valve sclerosis based on univariate logistic proportional regression analysis

GENE	OR	95% CI	P
RAN	1.044	1.025–1.063	.000
CCT7	1.049	1.026–1.073	.000
CSE1L	1.118	1.064–1.175	.000
SNRPD3	1.055	1.030–1.080	.000
TUBA1A	1.009	1.005–1.012	.000
USP14	1.155	1.067–1.251	.000
PSMD9	1.081	1.045–1.118	.000
CDK4	1.063	1.031–1.096	.000
RPL23A	1.010	1.006–1.014	.000
XRCC6	1.031	1.018–1.044	.000
EIF2S1	1.079	1.046–1.113	.000

**Table 9:** The hub genes and their effect on aortic valve sclerosis based on multivariate logistic proportional regression analysis

GENE	OR	95% CI	P
USP14	1.236	1.043–1.464	.015
XRCC6	1.033	1.005–1.062	.022

### Histological patterns of the aortic valve

**Fig. 7:**
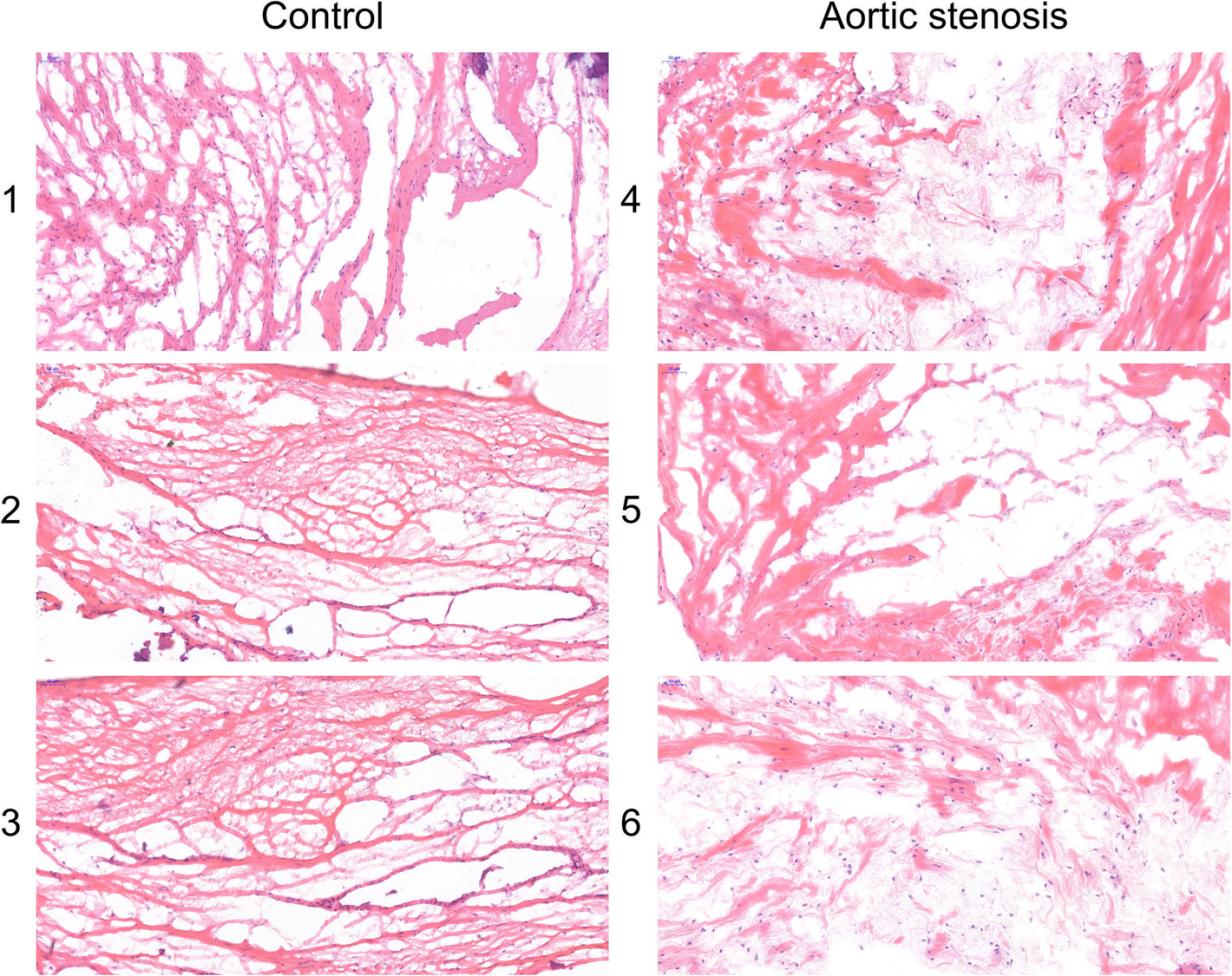
Histological patterns of aortic valve through HE staining

The HE staining demonstrated that the fibrous tissue and muscle cells were arranged regularly in the control aortic valve. However, the fibrous tissue and muscle cells were arranged irregularly in the aortic stenosis tissue (Fig. 7).

### The expression level of USP14 in the histology

**Fig. 8:**
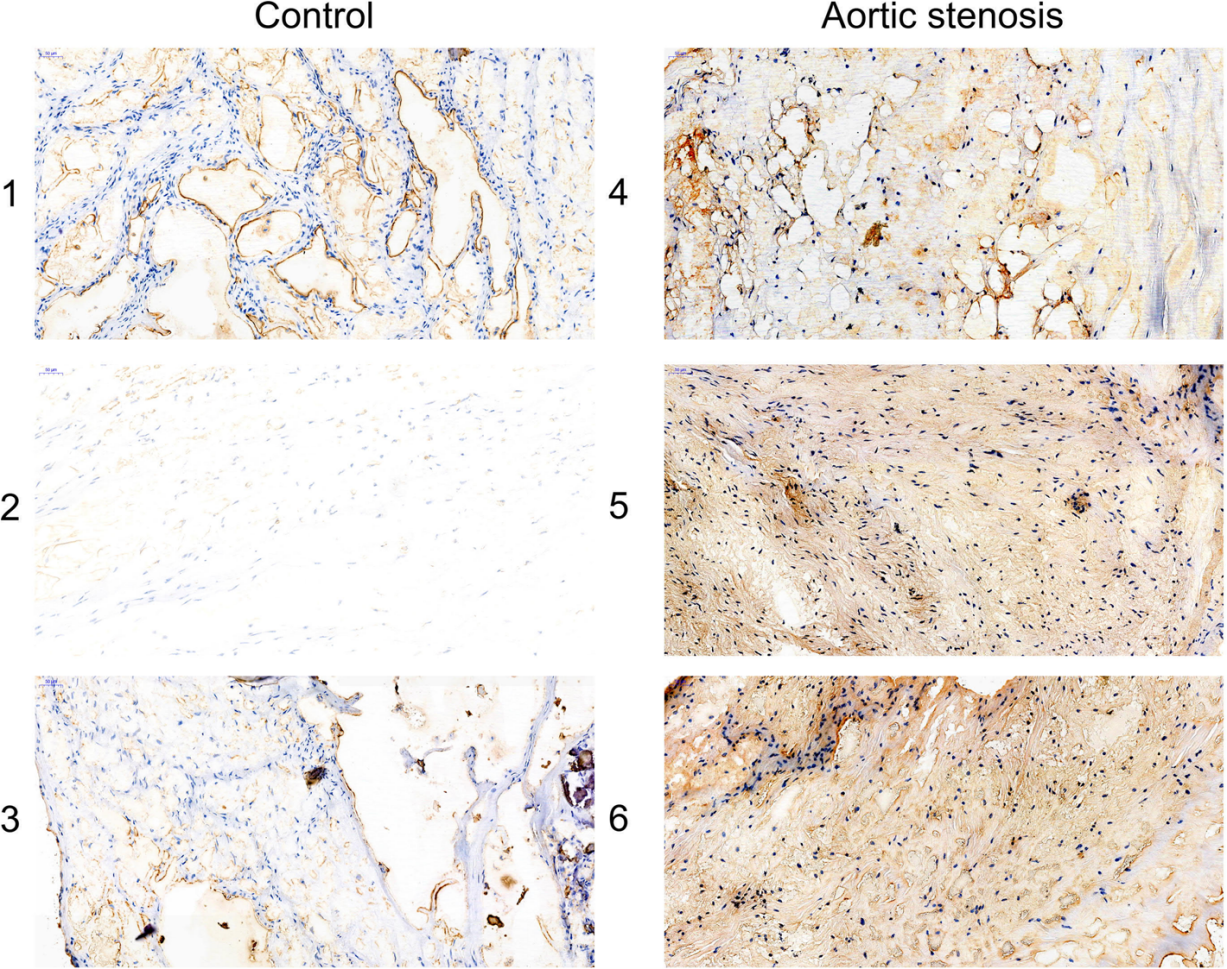
The expression level of USP14 in the histology via immunohistochemistry assay

**Fig. 9:**
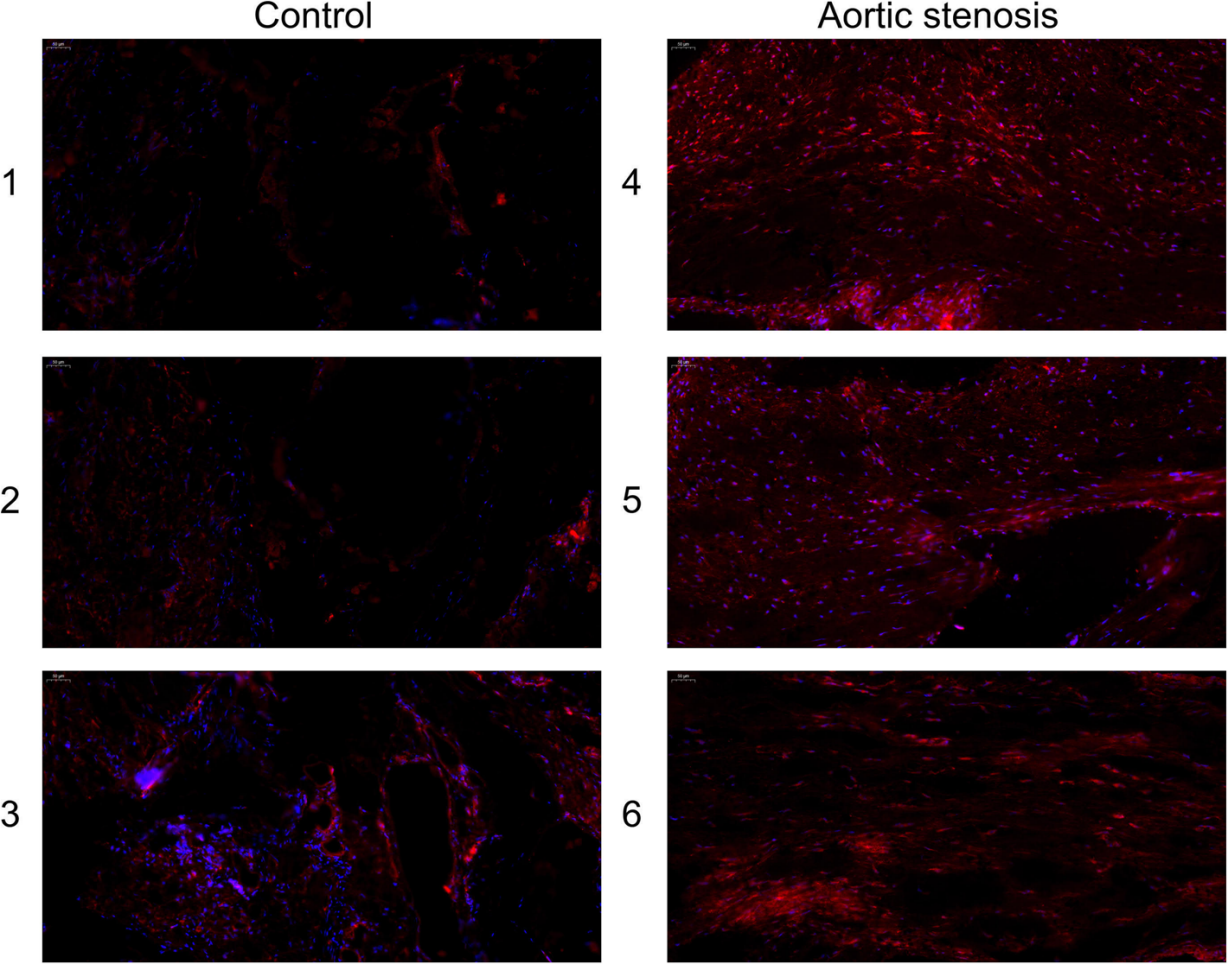
The detection of USP14 content of the aortic valve by immunofluorescence assay

The immunohistochemistry assay manifested that USP14 was upregulated in the aortic stenosis tissues, compared with the control aortic valve (Fig. 8). Also, an immunofluorescence assay corroborated such finding (Fig. 9).

### Verification of the expression of USP14 in the mRNA and protein levels

**Fig. 10:**
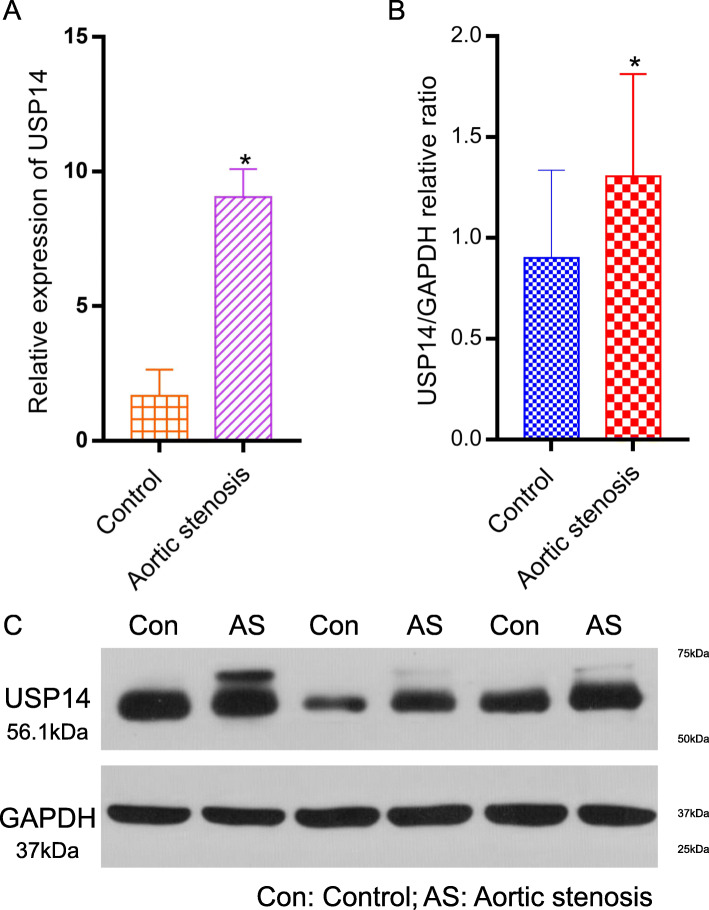
Verification of the expression of USP14 in the mRNA and protein level. **a** Relative expression of USP14 by RT-qPCR analysis. *p < 0.05, compared with control. **b**, **c** Western blotting expression of USP14 in the control and aortic stenosis groups

The RT-qPCR assay showed that the expression of USP14 was higher in the aortic stenosis than the control group (*P* < 0.05, Fig. 10a). Moreover, in terms of the protein levels, the Western blotting confirmed that USP14 was up-regulated in the aortic stenosis tissue compared with the control group (Figs. 10b, c). Western blotting corroborates immunohistochemistry and PCR findings.

## Discussion

In this study, a WGCNA on microarray data from aortic valves predicted that USP14 would be highly expressed in the valves of aortic stenosis patients, which was effectively verified in molecular experiments with samples collected from patients. USP14 may become a target for the diagnosis and treatment of aortic stenosis.

Aortic stenosis, a progressive and chronic disease, is one of the most serious cardiac valvular diseases [11]. In the sclerotic phase, aortic valves exhibit punctate calcification and valve thickening without hemodynamic changes and no obvious clinical symptoms, that is, aortic valve hardening. In the later stage, the valve ring and valve tissue appear to thicken, resulting in aortic valve stenosis and left ventricular outflow tract obstruction, which will eventually lead to serious obstruction of the left ventricular ejection function. The end stage of aortic stenosis is characterized by calcification, sided by the rupture of basement membranes, inflammatory cell infiltration, lipid deposition, endothelial dysfunction, and a series of activation processes caused by the transformation of valvular interstitial cells into osteoblasts [12].

USP14 is the only proteasome-binding deubiquitinating enzyme in the USP family. It can be recruited to 19 s particles to prevent it from being recognized and degraded by proteasome recognition, by cutting the ubiquitin chain on the protein substrate [13]. Accordingly, the inhibition of USP14 could improve the proteasome degradation activity [14]. USP14 is amplified and overexpressed in many types of diseases [15–18]. Overexpression of USP14 promotes cell proliferation and migration, while down-regulation induces cell apoptosis and inhibits cell proliferation, migration, and invasion. The expression of USP14 plays a pathogenic role in various types of diseases and is negatively correlated with the prognosis of the disease [19].

The coordination of the two reversible processes of ubiquitination and de-ubiquitination can finely regulate the type and degree of ubiquitination of proteins, affect the function and fate of corresponding proteins, and play an important role in many life activities such as cell division, differentiation, and signal transduction [20]. The USP14 protein has a total length of 494 amino acids and is divided into two domains: a ubiquitin-like domain (UBL) and a catalytic domain (CAT) [21]. It is one of the three deubiquitinating enzymes that bind to proteasome and can interact dynamically with proteins through ubiquitin. They regulate the function, metabolism, and degradation of proteins in the body, thus affecting a series of life activities such as the cell cycle, cell differentiation, transcription, DNA repair, immunity, and inflammation.

Dishevelled (Dvl) is a key regulator of Wnt signal transduction. USP14 can enhance the signal transduction of Wnt/β-catenin. The inhibition of USP14 can also increase the degree of ubiquitination of Dvl and significantly inhibit the downstream of Wnt signal transduction [18]. Therefore, the consequence of Wnt/β-catenin activation, due to USP14 Dvl de-ubiquitination, is that cell proliferation would be activated greatly, which would cause aortic valve hypertrophy and then lead to aortic stenosis. USP14 promotes the progression of cardiac hypertrophy by increasing GSK-3β phosphorylation [22]. This study found that USP14 is highly expressed in aortic valve tissue, which inhibits the proteasome degradation activity so this research hypothesizes that the inflammatory factors and lipoproteins gathered in the aortic valve cannot be effectively degraded, resulting in aortic stenosis.

Many studies have shown that the development of aortic stenosis is similar to atherosclerosis, which is a lipid inflammatory process [23]. However, the nature of the specific endogenous stimuli triggering aortic stenosis is not yet clear. Excessive activation of macrophages plays a very important role in inflammatory diseases. Under the stimulation of inflammatory factors or bacteria, the activation of macrophages produces more inflammatory cytokines, causing a cascade of inflammation, which eventually becomes uncontrolled inflammation.

Nuclear factor-κB (NF-κB) is an inducible transcriptional regulator of many inflammatory cytokine genes [24]. One study reported that overexpression of USP14 in lung epithelial cells can reduce IκB protein levels and increase cytokine release [25]. In addition, another study showed that USP14 affected NF-κB activation induced by lipopolysaccharide [26]. Based on these results, USP14 can induce the activation of NF-κB and regulate the up-regulation of tumor necrosis factor-α (TNF-α), interleukin-6 (IL-6), and interleukin-18 (IL-18) expression by activating the NF-κB signal pathway. These inflammatory factors interact to form a positive feedback loop to increase the production of inflammatory factors, aggravating the inflammatory response in the valve, promoting lipid deposition, and ultimately promoting the occurrence of aortic stenosis.

### Strength and study limitation

About the strength of the research, bioinformatics and clinical data were combined for analysis in this study. Many algorithms and experimental methods have been used to identify and verify the USP14 as an underlying gene biomarker for aortic stenosis. Although this study has carried out rigorous bioinformatic analysis, there are still some limitations. No animal experiments have been carried out. It is still necessary to further use an animal model to obtain more accurate results, in order to better understand the molecular mechanisms played by USP14 in aortic stenosis.

## Conclusions

In summary, bioinformatics technology could be one useful tool to predict the progression of aortic stenosis, by exploring the mechanism of its occurrence and development. DEGs, especially USP14, might be involved in the occurrence and development of aortic stenosis and might eventually become a biomarker and a target to treat this disease. Detection of USP14 in the clinical practice might provide the better evidence to guide the early diagnosis and treatment of aortic stenosis. In the future, diagnostic reagent kit of USP14 and the corresponding drugs should be developed to perform the multicenter randomized controlled clinical trial.

## Data Availability

The datasets used and/or analyzed during the current study are available from the corresponding author on reasonable request.

## Abbreviations

GEO: Gene Expression Omnibus
DEGs: Differently expressed genes
WGCNA: Weighted gene co-expression network analysis
PPI: Protein-protein interaction
HE: Hematoxylin-eosin
RT-qPCR: Real time-quantitative polymerase chain reaction
USP14: Ubiquitin-specific protease 14
AS: Aortic stenosis
AVR: Aortic valve replacement
TAVI: Transcatheter aortic valve implantation
FC: Fold change
GO: Gene Ontology
BP: Biological processes
CC: Cellular components
MF: Molecular functions
KEGG: Kyoto Encyclopedia of Genes and Genomes
GSEA: Gene Set Enrichment Analysis
CTD: Comparative toxicogenomics database
ROC: Relative operating characteristic
OR: Odds radio
PBS: Phosphate buffer solution
DAB: Diaminobenzidine
GAPDH: Glyceraldehyde-3-phosphate dehydrogenase
SDS-PAGE: Sodium dodecyl sulfate polyacrylamide gel electrophoresis
PVDF: Polyvinylidene fluoride
UBL: Ubiquitin-like domain
CAT: Catalytic domain
Dvl: Dishevelled
NF-κb: Nuclear factor-κB
TNF-α: Tumor necrosis factor-α
IL-6: Interleukin-6
IL-18: Interleukin-18
